# Effects of different silica intermediate layers for hydrogen diffusion enhancement of palladium membranes applied to porous stainless steel support

**DOI:** 10.1038/s41598-020-62054-3

**Published:** 2020-03-20

**Authors:** Masahiro Katoh, Tomoe Ueshima, Masahiro Takatani, Hikaru Sugiura, Kota Ominami, Shigeru Sugiyama

**Affiliations:** 1 Department of Applied Chemistry, Graduate School of Technology, Industrial and Social Sciences, Tokushima University, 2-1 Minamijosanjima-cho, Tokushima-shi, Tokushima 770-8506 Japan; 2 Department of Chemical Science and Technology, Tokushima University, 2-1 Minamijosanjima-cho, Tokushima-shi, Tokushima 770-8506 Japan

**Keywords:** Chemical engineering, Chemical engineering, Chemical engineering, Energy grids and networks, Energy grids and networks

## Abstract

Porous stainless steel (SUS) supports were modified with double intermediate layers, silicalite-1 and γ-alumina, to enhance the hydrogen diffusion of a thin palladium membrane. One of layers, silicalite-1, was prepared using the hydrothermal synthetic method on porous SUS supports. The differences in expansion/contraction behaviors caused by different thermal coefficients of expansion between silicalite-1 and the SUS resulted in a lowering of the durability of the membrane. Intermediates layers of mesoporous MCM-48 powders or commercial spherical non-porous silica particles were then applied to porous SUS supports via aspiration, γ-alumina was introduced by dip-coating, and the Pd membrane was subjected to electro-less plating. H_2_ permeance of the Pd membrane (membrane thickness: 11 μm) containing spherical silica particles was around 10 × 10^−6^ mol·m^−2^·s^−1^·Pa^−1^ at 600 °C, which was higher than that of the Pd membrane (membrane thickness: 7 μm) containing MCM-48. The durability of the Pd membrane containing spherical silica particles was higher than that of the version containing MCM-48 powders. These results suggest that commercial spherical non-porous silica particles will uniformly occupy the pores of the SUS tubes and enhance the H_2_ permeance and durability of the Pd membrane.

## Introduction

The synthesis of thin Pd membranes for high levels of hydrogen diffusion remains a difficult task. Thin Pd layers on porous supports provide mechanical stability. Ceramic supports have been extensively studied for use as porous supports. These ceramic supports, however, are quite brittle and difficult to integrate into metal housings, particularly under high temperatures^1,2^. Porous stainless (SUS) supports can be welded to non-porous SUS materials and easily assembled into modules, but intermetallic diffusion must be prevent at temperatures higher than 450 °C^3^. Two disadvantages of commercial porous SUS supports are a very wide pore size distribution and a high level of roughness. As a result, in order to prepare a defect-free Pd membrane, these SUS supports require a thickness equivalent that is three times the diameter of the largest pores^4^. The introduction of non-metallic intermediate layers can prevent intermetallic diffusion and smooth the surface of the supports. NaA zeolite^5^, NaX zeolite^6^, and siliceous materials^7^ were selected as the intermediate layer. The H_2_ permeance of a Pd membrane over a porous SUS tube with a non-metallic barrier, however, was insufficient for applications to membrane systems in hydrogen separation. In this study, we first focused on obtaining a Pd membrane with high H_2_ permeance. In our previous paper, we obtained a high level of H_2_/He selectivity with a pure Pd membrane over a porous SUS support. For example, the selectivity of the pure Pd membrane (Pd thickness: 34.6 μm) was 1,158^8^. After establishing a Pd membrane with high H_2_ permeance in the present work, the required level of hydrogen selectivity was obtained by increasing the Pd membrane thickness. In the present work, silicalite-1, MCM-48 powders, or commercial spherical silica particles and γ-alumina were selected as intermediate layers. In our previous reports, the alkali treatment of NaY zeolites^9^ and ZSM-5 zeolites^10^ enhanced their water diffusivity. Because silicalite-1 also has an MFI-type structure, alkali treatment was expected to enhance the gas diffusivity. A silicalite-1 layer hydrothermally applied over a porous SUS support was alkali-treated to enhance H_2_ diffusion. The alkali solution is usually used for Pd electro-less plating, which means it is very strong, and γ-alumina was selected to support the weak silicalite layer. We measured the levels of H_2_ permeance and H_2_/He selectivity obtained by Pd membranes over a porous SUS support with double intermediate layers (silicalite-1 and γ-alumina) and discussed the dependence that alkali conditioning had conferred. Three-dimensional mesoporous materials, MCM-48 powders, or commercial spherical non-porous silica particles were introduced to porous SUS supports via aspiration. MCM-48 was selected as powders that show high gas diffusivity via their three-dimensional mesoporous structure. Commercial spherical non-porous silica powders were selected for testing the necessity of pores for obtaining a high level of gas permeance. Gas would diffuse between silica particles. At that point, γ-alumina was coated onto MCM-48 or commercial spherical non-porous silica powders that were applied to a porous SUS support with double intermediate layers, and a Pd membrane was electro-less plated onto the double intermediate layers. We then examined the H_2_ permeance, H_2_/He selectivity, and the durability of the membranes.

## Experimental

Tubular porous stainless supports (PSS) with a media grade of 0.5 μm were provided by the Mott Corp. The supports had an external diameter of 1/2 inch (12.7 mm) and a length of 24 inches (609.6 mm). The original PSS supports were cut into 50 mm lengths.

### Preparing a palladium membrane with alkali-treated silicalite-1 and γ-alumina layers to cover a porous SUS tubular support

The first step in the preparation of palladium membranes involved the cleaning of PSS supports^11^, which was then followed by four successive steps performed over the cleaned supports: i) incorporation of a silicalite-1 intermediate layer, ii) alkali treatment of a silicalite-1 intermediate layer, iii) incorporation of a γ-alumina intermediate layer, and, iv) palladium deposition.

#### Synthesis of a Silicalite-1 Seed crystal

The silicalite-1 seed crystals were prepared via hydrothermal synthesis^12^. The starting solution consisted of a template of tetra-*n*-propylammonium bromide (TPABr), colloidal silica (Cataloid SI-30, JGC Catalysts and Chemicals Ltd.), sodium hydroxide, and distilled water in a molar ratio of.

$${{\rm{SiO}}}_{2}:{\rm{NaOH}}:{\rm{TPABr}}:{{\rm{H}}}_{2}{\rm{O}}=1:0.2:0.1:40.$$

This hydrogel was stirred at 40 °C for 24 h. Subsequently, the hydrogel was hydrothermally treated at 100 °C for 6 days under 1 atm using an open-system unit. The obtained crystals were washed with distilled water and calcined at 550 °C for 6 h in order to eliminate the tetra-*n*-propylammonium cation (TPA^+^) that remained in the silicalite-1 pores. The crystal structure was analyzed using X-ray diffraction microscopy (XRD, SmartLab9kW; RIGAKU Corp.). The morphology was observed with a scanning electric microscope (SEM, JSM-6390HV; JEOL Ltd.). The particle size distribution was measured using an electrophoretic light scattering spectrometer (Microtrac Version 10.4.0–225 F; Nikkiso. Co.).

#### Deposition of seed crystals on a porous stainless steel support

The 130 millimeter-long PSS support had a porous length of 50 mm, a non-porous lengths of 79 mm, and a 1 mm lid. The support was immersed in a seed crystal aqueous solution (30 g/L) for 1 h and was dried at 110 °C.

#### Preparation of the Silicalite-1 layer

The silicalte-1 layer was prepared via hydrothermal synthesis^12^. The starting hydrogel for the synthesis of the silicalite-1 layer consisted of TPABr as a template, colloidal silica, sodium hydroxide, and distilled water in a ratio of SiO_2_:NaOH:TPABr:H_2_O = 1:0.05:0.0025:80. This hydrogel was stirred at room temperature for 2 h and placed in an autoclave. In addition, a seeded tubular support was also vertically placed in the autoclave. The hydrothermal synthesis was carried out at 170 °C for 48 h. The obtained layer was repeatedly washed with distilled water, dried at 110 °C for 24 h, and calcined at 550 °C for 6 h to eliminate the TPA^+^.

#### Alkali treatment of the silicalite-1 layer

The silicalite-1 layer was treated with 0.01 M, 0.02 M, 0.03 M, and 0.2 M aqueous solution of NaOH^9,10^. The silicalite-1 layer was placed into a 250 mL NaOH solution that was maintained at 80 °C for 30 min with constant stirring. After the alkali treatment, the membrane was washed in distilled water at 80 C for 2 h with constant stirring. Finally, the membrane was allowed to dry at 110 °C for 24 h.

#### Placing a γ-alumina layer onto the alkali-treated silicalite-1 layer

An alumina layer was placed onto the alkali-treated silicalite-1 layer to protect it from the destruction by the high pH of the Pd plating solution. The alumina layer was dip coated with a Boehmite sol^13^. The Boehmite sol was prepared by hydrolyzing 1.531 g of aluminum isopropoxide (Wako Pure Chemicals, First grade) in 150 mL of distilled water at 80 °C for 24 h in a four-necked, round-bottomed flask equipped with a reflux condenser and three stoppers. After hydrolysis, these stoppers were removed from the flask, and the alcohol was allowed to evaporate from the solution at 90 °C over 4 h. After the flask was again stoppered for 1 h, we then added 0.06 mL HNO_3_ (Wako Pure Chemicals, Special grade) to the solution to promote peptization of the hydroxide at 80 °C for 24 h to form a Boehmite sol.

The alkali-treated silicalite-1 layer was dipped into the Boehmite sol for 10 s and pulled up at 1.0 cm/s. The layer coated with the sol was then dried at room temperature in a desiccator for 24 h. The coated layer was kept in an oven wherein the temperature was increased by 1 °C/min until a calcination temperature of 600 °C was reached. After the layer was calcined at this temperature for 3 h, the temperature was decreased by 1 °C/min until it reached room temperature. After calcination, the γ-alumina layer was adhered to an alkali-treated silicalite-1 layer. The dip-coating and calcination steps were repeated five times. In this study, three kinds of siliceous materials were chosen. Although the hydrophobic properties depended on the materials, these materials were coated by the same γ-alumina. Pd plating was performed on γ-alumina with hydrophilicity under the following procedures.

#### Palladium plating the γ-alumina layer onto the alkali-treated silicalite-1 layer

Once the supports were coated with double intermediate layers (γ-alumina layer and an alkali-treated silicalite-1 layer), they were activated and plated with palladium metal via electro-less deposition^8,11,14^. The activation procedure involved successive immersions of the supports in an acidic SnCl_2_ bath and an acidic PdCl_2_ bath. After the immersion in the SnCl_2_ bath, the supports were gently rinsed with deionized water. After immersion in the PdCl_2_ bath, the supports were rinsed with HCl and then with distilled water. Pd deposition was accomplished according to the following reaction.

$$2{\rm{Pd}}{({{\rm{NH}}}_{3})}_{4}{{\rm{Cl}}}_{2}+{{\rm{H}}}_{2}{{\rm{NNH}}}_{2}+4{{\rm{NH}}}_{4}{\rm{OH}}\to 2{\rm{Pd}}+{{\rm{N}}}_{2}+8{{\rm{NH}}}_{3}+4{{\rm{NH}}}_{4}{\rm{Cl}}+4{{\rm{H}}}_{2}{\rm{O}}$$

The thickness of the Pd layer was calculated by the amount of plated Pd and the density of the Pd (12.0 g/cm^3^).

### Preparation of a palladium membrane made up of MCM-48 and γ-alumina layers covering a porous SUS tubular support

The preparation of palladium membranes began with the cleaning of the PSS supports^11^ followed by three successive steps performed on the cleaned supports: i) incorporation of a MCM-48 intermediate layer, ii) incorporation of a γ-alumina intermediate layer, and, iii) palladium deposition.

#### Synthesis of MCM-48 powders

The MCM-48 powders were prepared based on a procedure established in a previous report^15^. The starting solution consisted of hexadecyltrimethylammonium bromide (CTAB; KANTO Chemical Co. INC.) as the template, aq. NH_3_, Tetraethyl orthosilicate (TEOS; KANTO Chemical Co. INC.), ethyl alcohol (ETOH; Wako Pure Chemicals), and distilled water. The solution consisted of the following molar ratio:

$${\rm{CTAB}}:{\rm{aq}}.{{\rm{NH}}}_{3}:{\rm{TEOS}}:{\rm{EtOH}}:{{\rm{H}}}_{2}{\rm{O}}=0.41:11:1.0:53:344.$$

This hydrogel was stirred at room temperature for 2 h. The obtained white powders were washed with distilled water, dried at 100 °C for 24 h and calcined at 550 °C for 6 h in order to eliminate the hexadecyltrimethylammonium cation (CTA^+^) that remained in the MCM-48 pores. Particle size distribution of the obtained materials showed broad peaks ranging from 0.1 to 100 μm. Both the Pd membrane over these particles and the γ-alumina layers sometimes fell off, which indicated that variety in the size of the the MCM-48 particles lowered the membrane strength. As a result, calcined MCM-48 powders were crushed lightly in a mortar and showed a uniformly distribution (ca. 0.8 μm) (see Fig. S.1). The crystal structure of the obtained MCM-48 was checked using X-ray diffraction microscopy (XRD, SmartLab9kW; RIGAKU Corp.). The morphology was observed using a scanning electric microscope (SEM, JSM-6390HV; JEOL Ltd.). The particle size distribution was measured using an electrophoretic light scattering spectrometer (Microtrac Version 10.4.0–225 F; Nikkiso. Co.).

#### Introduction of MCM-48 powders into porous SUS supports; coating the supports with an γ-alumina layer; and, palladium plating of the porous supports

MCM-48 powders (0.03 g) were dispersed into 450 mL of distilled water via ultrasonic bath. The dispersed powders were introduced into porous SUS supports via aspiration. The aspiration was repeated twice. γ-alumina was then coated onto the supports, and Pd membranes were attached to the porous SUS supports that already had double intermediate layers. These procedures were described in sections 2.1.5 and 2.1.6.

### Attaching the palladium membrane to the commercial spherical non-porous silica particles and γ-alumina layers covering the porous SUS tubular supports

The preparation of palladium membranes began with the cleaning of the PSS supports^11^ followed by three successive steps performed over the cleaned supports: i) incorporation of an intermediate layer of commercial spherical silica particles, ii) incorporation of an γ-alumina intermediate layer, and, iii) palladium deposition.

Non-porous amorphous silica particles with a diameter 1 of μm (SEAHOSTER; KE-S100) were supplied by NIPPON SHOKUBAI Co., LTD. The particles were selected according to the size of MCM-48, as shown in Section 2.2.1. Then, 0.03 g of silica powder was dispersed into 450 mL of distilled water via ultrasonic bath. All of the dispersed powder was introduced to a porous SUS support via aspiration. The aspiration was repeated twice. Next, γ-alumina was coated onto the SUS supports followed by attachment of a Pd membrane. These procedures are fully described in sections 2.1.5 and 2.1.6.

### Permeation experiments

**Figure 1:**
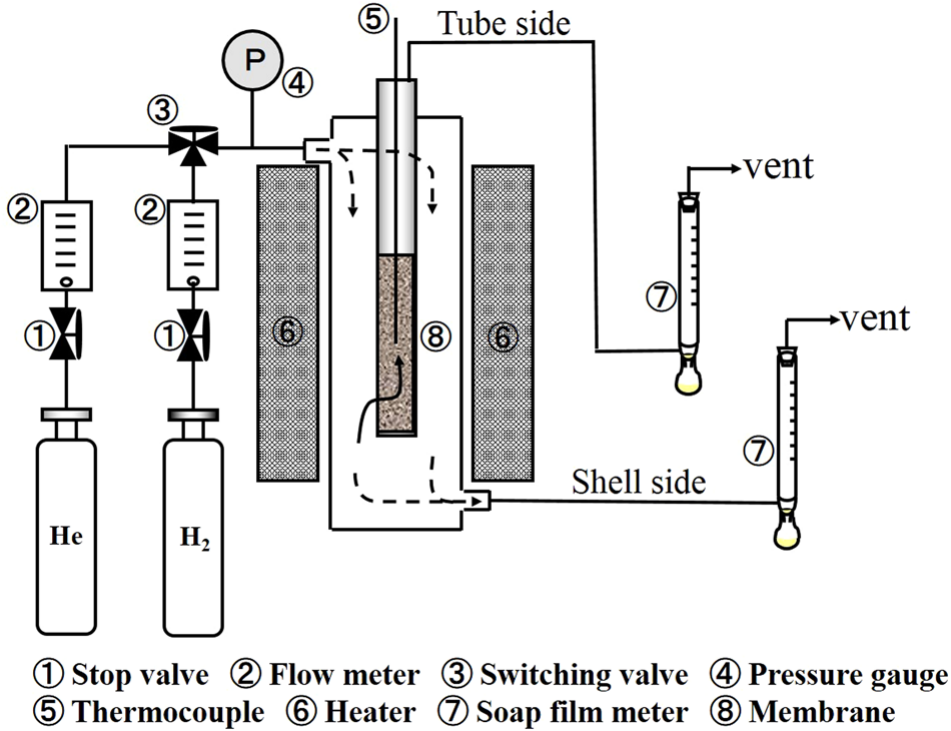
Experimental setup for permeation measurements.

The H_2_ or He permeance for obtained Pd membranes with alkali treated silicalte-1, MCM-48, or commercial spherical silica powders introduced into the porous SUS support was measured by using a shell-and-tube membrane module shown in Fig. 1. The membrane was sealed using graphite O-ring^8^. The operating temperature was measured using a K-type thermocouple inserted in the membrane unit. The pressure of the flowing gas was monitored with a pressure gauge. The pressure of the permeate-side maintained at atmospheric pressure and no sweep gas was used. The permeate flux was measured using a soap film meter maintained at ambient conditions (i.e. atmospheric pressure and room temperature). After H_2_ permeation test, the chemical valences of the Pd species of the Pd membrane checked by XPS characterization, as described in our previous paper^8^. The Pd 3d XPS spectra for the surface of the Pd membrane detected only Pd(0). The chemical valences of the Pd species on surface of Pd membrane showed was not affected by H_2_ permeation.

The H_2_ flux was measured at temperatures greater than 300 °C to avoid H_2_ embrittlement^16^. Before the H_2_ permeation tests were performed, the membrane was heated in He at a rate of about 1 °C/min. In the absence of purging, a negative flux would have been observed as a result of back diffusion of the dissolved H_2_. The purities of H_2_ and He were 99.95 and 99.99%, respectively. Experimental temperatures were 400, 500, 600 °C, and the differential pressure, ΔP, was 0.1 MPa.

The ideal H_2_/He selectivity is defined as the ratio of the permeance (J) of the two pure gases, as follows:

1 $${{\rm{H}}}_{2}/{\rm{He}}\,{\rm{selectivity}}=\frac{{J}_{{H}_{2}}}{{J}_{He}}$$

### Testing the durability of H2 permeation using a temperature cycle

The durability test for H_2_ permeation was done for three obtained Pd membranes with alkali treated silicalte-1, MCM-48, or commercial spherical silica powders introduced into the porous SUS support. The total H_2_ permeation time was 50 h. A temperature cycle from 600 °C to room temperature and from room temperature to 600 °C was added to the durability test under a He atmosphere to avoid H_2_ embrittlement.

## Results and Discussion

### The structure of silicalte-1 seed crystals

**Figure 2:**
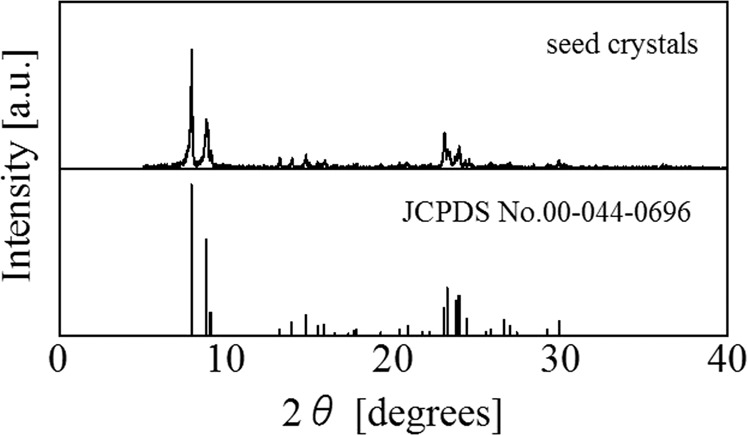
XRD patterns of silicalite-1 seed crystals and the reference.

**Figure 3:**
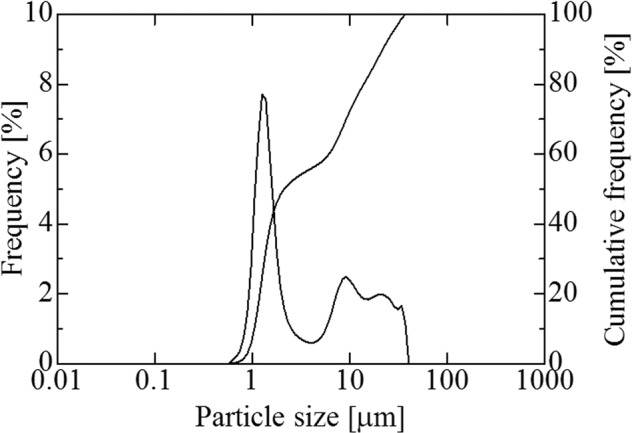
Particle size distribution of silicalite-1 seed crystals.

**Figure 4:**
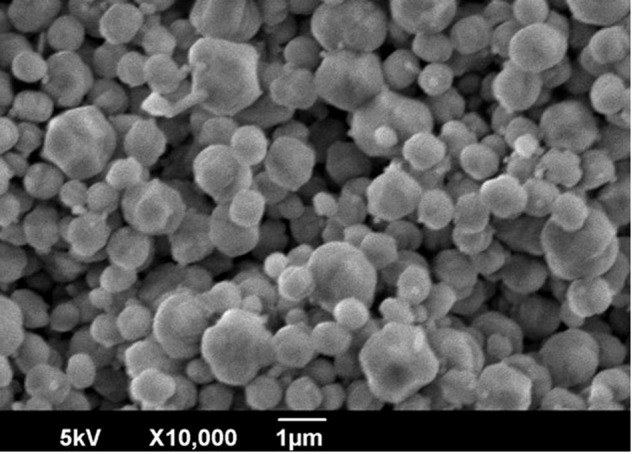
SEM image of silicalite-1 seed crystals.

Figure 2 shows the XRD patterns of silicalite-1 seed crystals and a reference by JCPDS. The XRD pattern of the prepared crystals almost matched the pattern of the reference. The particle size distribution of the prepared crystals appears in Fig. 3, and a SEM image of the prepared crystals appears in Fig. 4. Figure 3 shows a large sharp peak (mode size: 1.26 μm) and two small peaks (around 9.25 μm). Because large particles of approximately 10 μm did not appear in the SEM image, we concluded that the peaks of a larger particle size distribution represented aggregates. The prepared silicalite-1 seed crystals reached a size of approximately 1 μm.

### He permeance of the Pd membrane with γ-alumina and silicalte-1 layers

**Figure 5:**
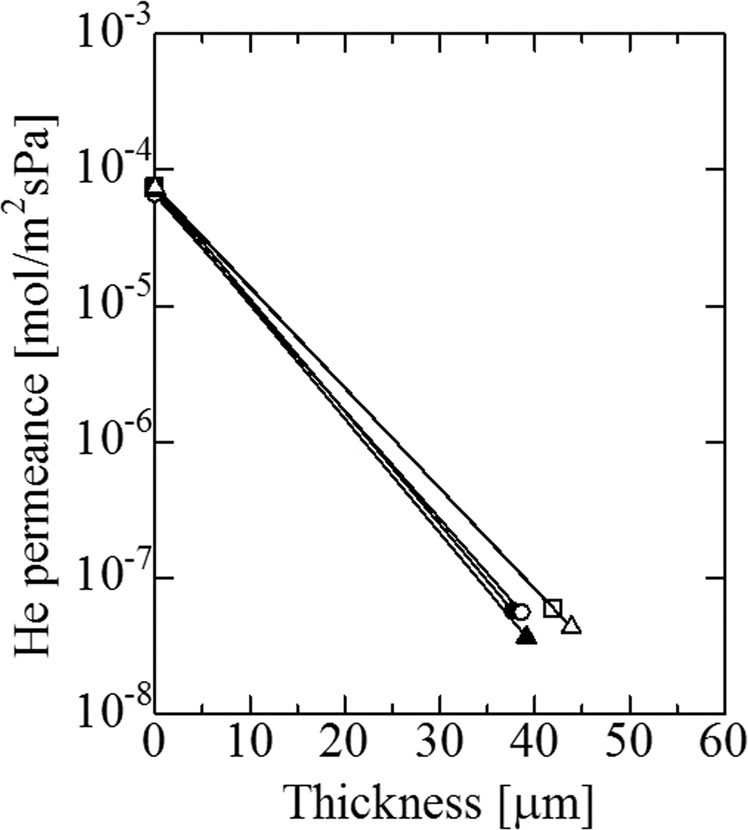
Change of He flux to the thickness of the silicalite-1 layer at room temperature before and after coating with silicalite-1 layer (ΔP = 0.1 MPa).

**Figure 6:**
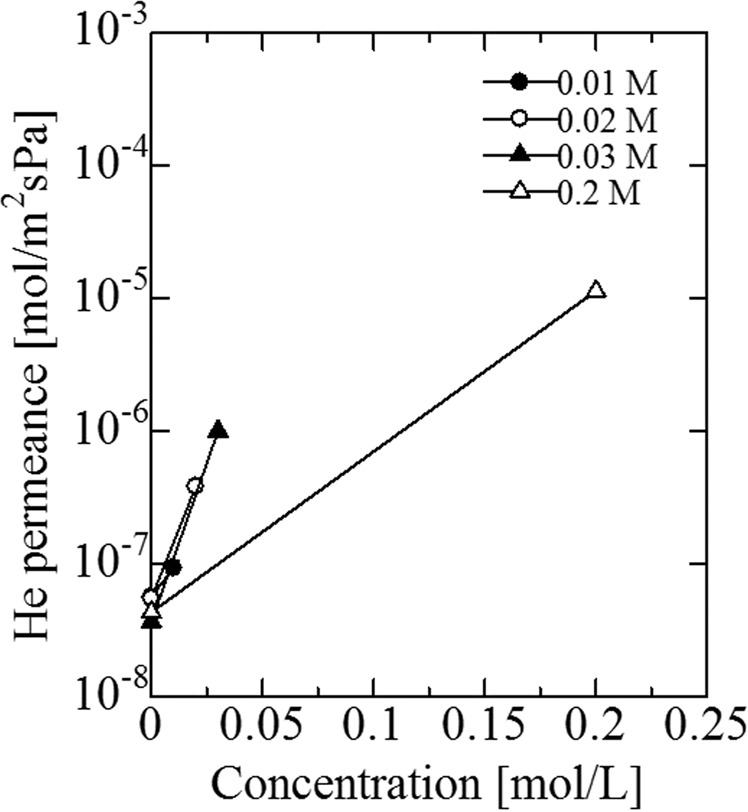
Change of He flux to concentration of sodium hydroxide aqueous solution at room temperature before and after alkali treatment (ΔP = 0.1 MPa).

**Figure 7:**
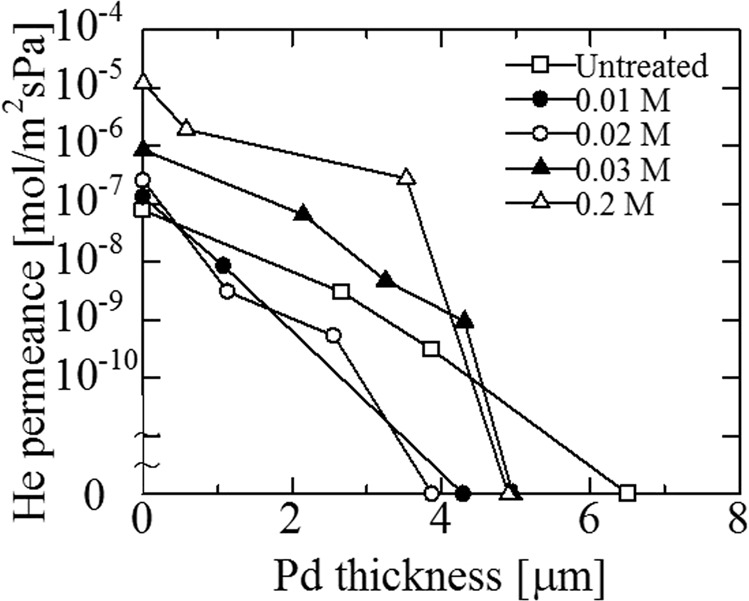
Change of He flux to Pd thickness for Pd membrane on the double intermediate layers (alkali treated silicalite-1 and γ-alumina layers) at room temperature (ΔP = 0.10 MPa).

Before alkali treatment, He permeance was changed by the preparation of the silicalite-1 layer, as shown in Fig. 5. He permeance of the five porous SUS supports, after modification with a silicalite-1 layer, was decreased from approximately 10^−4^ mol·m^−2^·s^−1^·Pa^−1^ to less than 10^−7^ mol·m^−2^·s^−1^·Pa^−1^. Reproducible results were obtained for the five supports. The results showed that the large pores of the SUS supports were almost closed by the silicalite-1 layer. The changes in He permeance following alkali treatment are illustrated in Fig. 6. He permeance was increased by the alkali treatment, which increased with the concentration of the alkali treatment. These results indicated the diffusivity of He in the pores of the silicalite-1 layer, which was enhanced by the alkali treatment. The changes in He permeance via Pd plating are shown with the presence of the double intermediate layers (alkali treated silicalite-1 and γ-alumina) in Fig. 7. He permeance was suddenly decreased to a value that reached 0 following Pd plating.

### H_2_ and He permeance of the Pd membrane with γ-alumina and alkali-treated silicalte-1 layers

**Figure 8:**
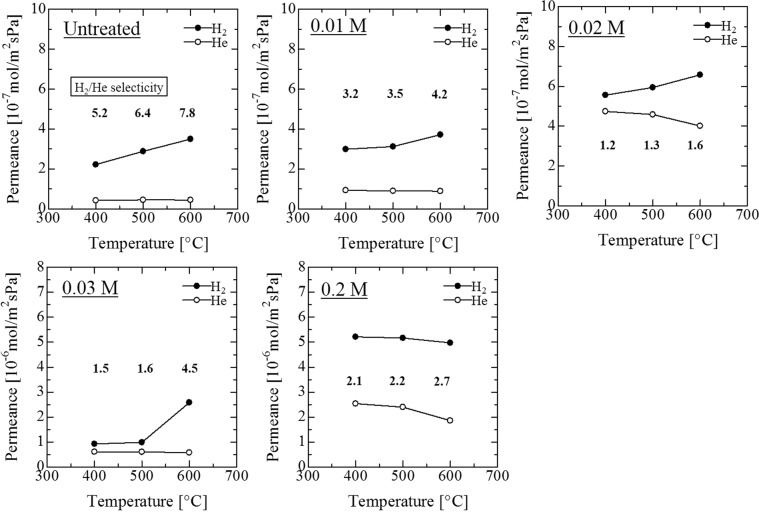
Temperature dependence of H_2_ and He permeance of Pd membranes on porous SUS tubes with double intermediate layers (alkali treated silicalite-1 layer at each concentration and γ-alumina layer) (ΔP = 0.10 MPa).

The diffusion mechanism of H_2_ in the Pd membrane was the solution diffusion mechanism and that of He in the membrane was Knudsen diffusion and viscos or Poiseuille flow^11^. Figure 8 shows the temperature dependence of H_2_ and He permeance after the Pd plating of the Pd membrane onto porous SUS tubes with double intermediate layers of alkali-treated silicalite-1 and γ-alumina (ΔP = 0.10 MPa) at each concentration. H_2_ permeance at 600 °C was smaller than 7 × 10^−7^ mol·m^−2^·s^−1^·Pa^−1^, which is below that for 0.02 M of a NaOH aqueous solution. But He permeance at 600 °C was higher than the 1.5 × 10^−6^ mol·m^−2^·s^−1^·Pa^−1^ recorded for 0.2 M of a NaOH aqueous solution. As the results show, the optimal concentration for a NaOH aqueous solution was determined. The Pd membrane plated onto a silicalite-1 layer that had been alkali-treated by a 0.03 M NaOH aqueous solution showed the highest level of H_2_ permeance at more than 2.5 × 10^−6^ mol·m^−2^·s^−1^·Pa^−1^ for all the prepared samples, and also recorded the highest selectivity level of H_2_/He = 4.5 at 600 °C for all alkali-treated samples.

### Durability of H_2_ permeation for a Pd membrane covering porous SUS supports impregnated with double intermediate layers (0.03 M alkali treated silicalite-1 and γ-alumina)

**Figure 9:**
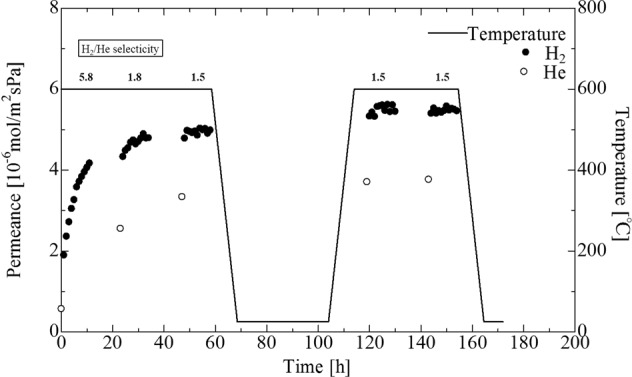
Time dependence of H_2_ or He permeance of Pd membrane on porous SUS tube with double intermediate layers (alkali treated (0.03 M NaOH solution) silicalite-1 and γ-alumina layers) (ΔP = 0.1 MPa).

Figure 9 shows the time dependence of the H_2_ and He permeance of a Pd membrane covering porous SUS tubes impregnated with double intermediate layers of silicalite-1 and γ-alumina (ΔP = 0.1 MPa). In this test, the measurement of H_2_ permeance was performed 10 h x 5 times. He permeated the membrane when the test temperature was changed from 600 °C to room temperature and from room temperature to 600 °C. The first reading for H_2_/He selectivity was 5.8, but H_2_ permeance decreased the selectivity to 1.5 after 50 h. This was induced by an increase in the permeance of He, which indicated the generation of a leak from the Pd membrane. Differences in the expansion/contraction behaviors between silicalite-1 and SUS^17^ due to differences in the thermal coefficients of expansion resulted in a low level of durability for the membrane. In the present study, the silicalite-1 seed crystals were seeded onto a SUS support, and the silicalite-1 layer was then synthesized via a hydrothermal method. The high level of adhesion between the silicalite-1 layer and the SUS lowered the membrane durability. In order to decrease adhesion between the intermediate layers and the SUS, either our synthesized mesoporous silica; MCM-48 powders, or commercial spherical silica particles were applied to the porous SUS supports.

### The structure of MCM-48 powders and commercial spherical non-porous silica particles

After the MCM-48 powders were crushed lightly in a mortar, a SEM image (see Fig. S.1) was acquired and the particle distribution (see Fig. S.2) was measured. This evidence showed that of the MCM-48 powders were made up of particles with an average size of 0.8 μm and were not spherical.

A SEM image (see Fig. S.3) was acquired for commercial spherical non-porous silica particles, and the particle distribution (see Fig. S.4) was measured. These spherical particles had an average particle diameter of 1 μm.

### He permeance of a Pd membrane mounted on γ-alumina and MCM-48 powders or commercial spherical non-porous silica particles layers

**Figure 10:**
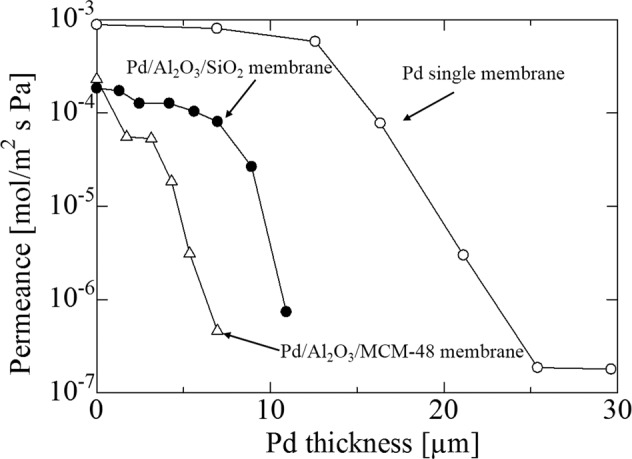
Change of He flux to Pd thickness for Pd membrane on the non-intermediate layer and double intermediate layers (MCM-48 layer or spherical commercial silica particles layer and γ-alumina layer) at room temperature (ΔP = 0.10 MPa).

He permeance was changed by Pd plating, as shown in Fig. 10, with a non-intermediate layer in a Pd single membrane, double intermediate layers of MCM-48 and γ-alumina for Pd/Al_2_O_3_/MCM-48 membranes, and double intermediate layers of commercial spherical non-porous silica particles and γ-alumina for Pd/Al_2_O_3_/SiO_2_ membranes at room temperature (ΔP = 0.10 MPa). He permeance for a Pd single membrane reached less than 10 × 10^−6^ mol·m^−2^·s^−1^·Pa^−1^ following Pd plating with a 25 μm membrane thickness. He permeance was not changed by additional Pd plating. These results showed that the pores of the SUS supports were almost closed by the Pd plating. Pd plating was performed on the double intermediate layers until He permeance levels fell to less than 10 × 10^−6^ mol·m^−2^·s^−1^·Pa^−1^. He permeance fell to less than 10 × 10^−6^ mol·m^−2^·s^−1^·Pa^−1^ with Pd plating that reached a membrane thickness of 7 μm for a Pd/Al_2_O_3_/MCM-48 membrane and 11 μm of membrane thickness for a Pd/Al_2_O_3_/SiO_2_ membrane. A thin palladium membrane that compared favorably to a Pd single membrane was obtained by introducing double intermediate layers into a porous SUS support.

### H_2_ permeance and H_2_/He selectivity for Pd membranes with no intermediate layer, with γ-alumina and MCM-48 intermediate layers, and with γ-alumina and spherical commercial non-porous silica intermediate layers

**Figure 11:**
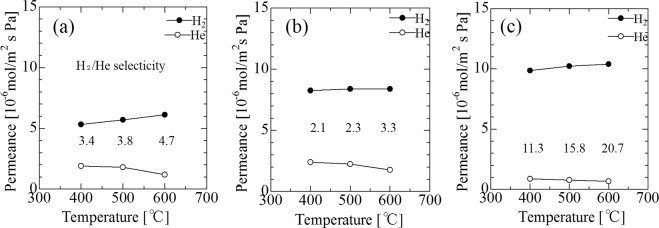
Temperature dependence of H_2_ and He permeance of Pd membrane on the non-intermediate layer (Pd single membrane) (**a**), double intermediate layers (MCM-48 and γ-alumina layers) (Pd/Al_2_O_3_/MCM-48 membrane) (**b**) and double intermediate layers (spherical commercial silica particles and γ-alumina layers) (Pd/Al_2_O_3_/SiO_2_ membrane) (**c**) (ΔP = 0.10 MPa).

Figure 11 showed the temperature dependence of H_2_ and He permeance for Pd membranes mounted on porous SUS tubes without (Pd single membrane) or with double intermediate layers (Pd/Al_2_O_3_/MCM-48 membrane and Pd/Al_2_O_3_/SiO_2_ membrane) (ΔP = 0.10 MPa). For MCM-48 powder-induced membrane, the He permeance was higher than that of spherical commercial non-porous SiO_2_ particle-induced membrane. On the other hand, the H_2_ permeance for spherical SiO_2_ particle- induced membrane was higher that of MCM-48 powder-induced membrane. The reasons are discussed in Section 3.8. Of the three membranes, the highest H_2_ permeance (10 × 10^−6^ mol·m^−2^·s^−1^·Pa^−1^) and H_2_/He selectivity (20.7) was recorded for the Pd/Al_2_O_3_/SiO_2_ membrane at 600 °C. This membrane’s H_2_ permeance was much higher than the Pd membrane over the support modified with silicalite-1 (3.16 × 10^−7^ mol·m^−2^·s^−1^·Pa^−1^)^7^. This membrane’s selectivity was much lower than the reference’s membrane. However, the required level of hydrogen selectivity could be obtained by increasing the Pd membrane thickness. In comparison to the Pd single membrane, the higher level of H_2_ permeance for Pd membranes with double intermediate layers was induced by thinner Pd version.

### Durability test for H_2_ permeation of the Pd membrane with double intermediate layers (γ-alumina and MCM-48 layers or γ-alumina and spherical commercial non-porous silica layers) on porous SUS supports

**Figure 12:**
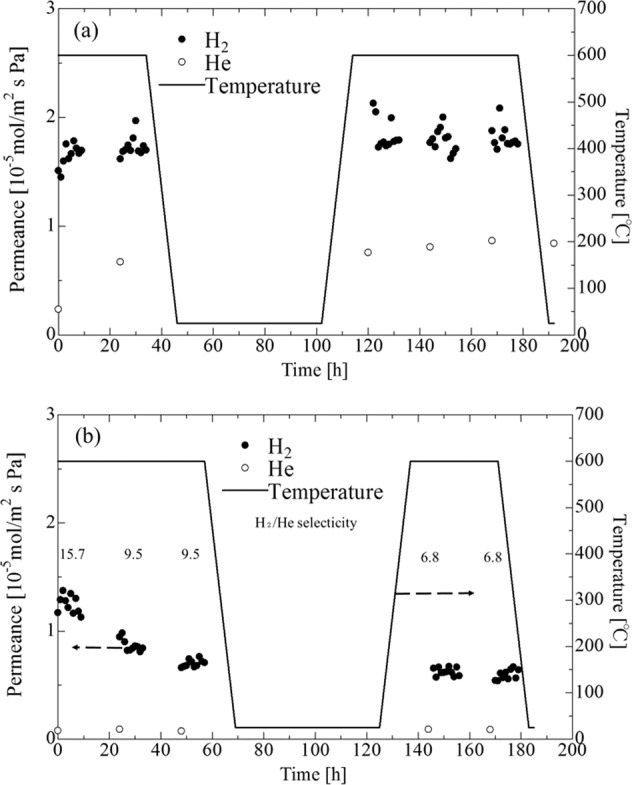
Time dependence of H_2_ or He permeance of Pd membrane for Pd/Al_2_O_3_/MCM-48 membrane (**a**) and Pd/Al_2_O_3_/SiO_2_ membrane (**b**) on porous SUS tube (ΔP = 0.1 MPa).

Figure 12 shows the time dependence for both H_2_ and He permeance of a Pd membrane affixed to a porous SUS tube with double intermediate layers (Pd/Al_2_O_3_/MCM-48 membrane (a) and Pd/Al_2_O_3_/SiO_2_ membrane (b)) (ΔP = 0.1 MPa). The Pd/Al_2_O_3_/MCM-48 membrane first showed a H_2_/He selectivity of 7.0, which decreased to 2.1 for H_2_ permeance after 50 h. This was induced by an increase in the He permeance, which suggested the generation of a leak in the Pd membrane. On the other hand, for the Pd/Al_2_O_3_/SiO_2_ membrane, although H_2_ permeance was decreased over the first 30 h, the value remained stable after the temperature cycle and the He permeance was unchanged after 50 h. As a result, the H_2_/He selectivity of 6.8 was preserved after the temperature cycle.

**Figure 13:**
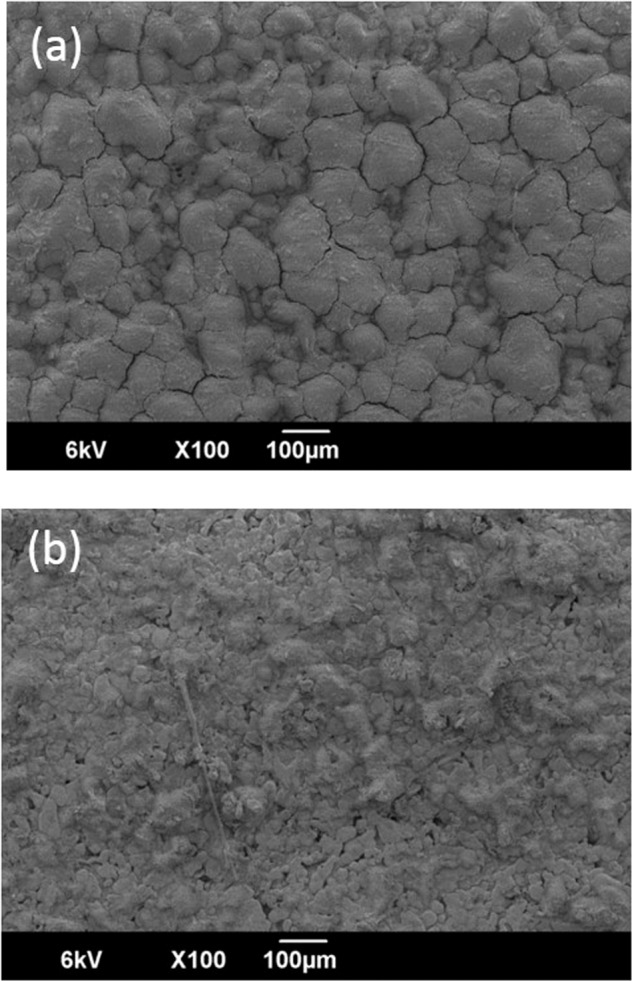
SEM images of Al_2_O_3_/MCM-48 layers (**a**) and Al_2_O_3_/SiO_2_ layers (**b**).

**Figure 14:**
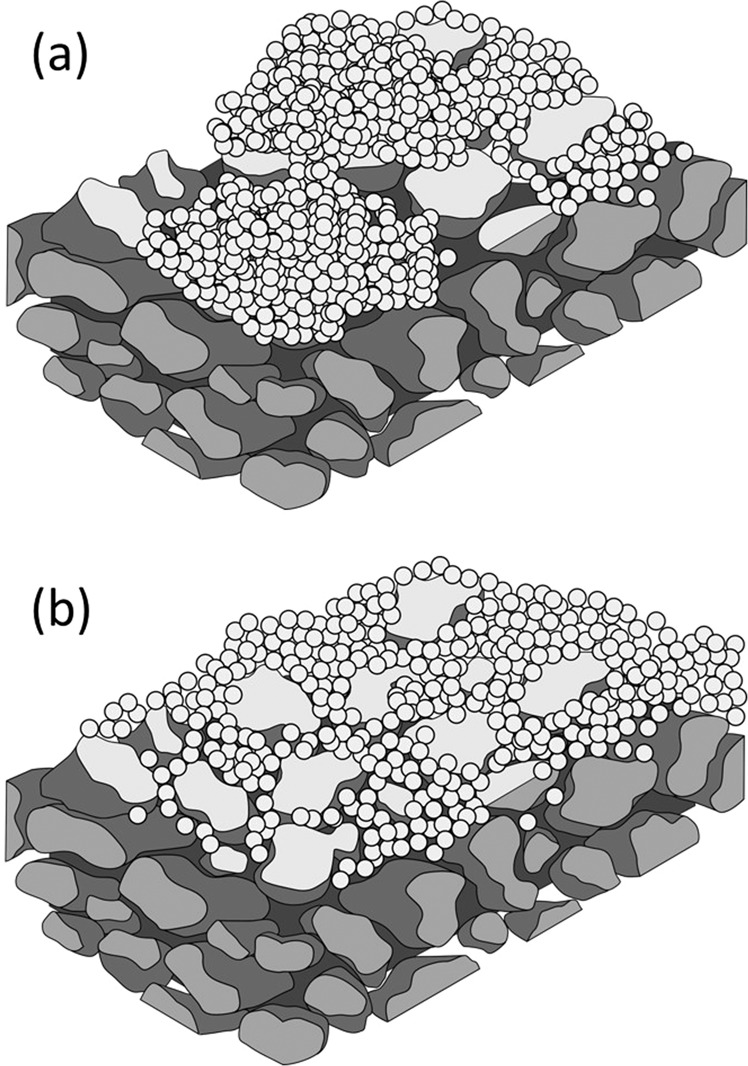
Images of Al_2_O_3_/MCM-48 layers (**a**) and Al_2_O_3_/SiO_2_ layers (**b**).

To document the differences in the permeance behaviors between the Pd/Al_2_O_3_/MCM-48 and Pd/Al_2_O_3_/SiO_2_ membranes, we measured the SEM images of these double intermediate layers before Pd plating (Fig. 13). Although the amount of MCM-48 or non-porous silica layers introduced was the same, 0.06 g (0.03 g × 2), their morphologies were different. The SEM images of the Al_2_O_3_/MCM-48 layers on the porous SUS supports showed aggregates of MCM-48 covered all of the SUS support, but the pores of the SUS supports were not completely occupied by the MCM-48 powders. Insufficient introduction of the powders into the pores of the SUS supports induced low durability values for H_2_ permeance by the membrane after 50 h. On the other hand, when the Al_2_O_3_/SiO_2_ layer was introduced to the porous SUS supports, the non-porous silica particles filled the pores of SUS supports. The commercial spherical non-porous silica particles uniformly occupied the pores of the SUS tubes, and both the H_2_ permeance and the durability of the Pd membrane were enhanced. The surface morphology also could explain the He and H_2_ permeance performance. The Pd membrane plated on the rough surface generated by the aggregates of MCM-48 showed lower levels of H_2_ permeance. On the other hand, the uniform Pd membrane generated by silica particles showed higher level of H_2_ permeance. The higher He permeance might have been induced by the three-dimensional mesopore structure. However, a higher level of H_2_ permeance was achieved by the H_2_ diffusion between non-porous SiO_2_ particles. These results appear herein as Fig. 14.

## Conclusions

This study represents an investigation into the preparation of a thin palladium membrane for hydrogen diffusion enhancement using porous stainless steel (SUS) supports modified via the introduction of double intermediate layers (alkali-treated siliclalite-1, MCM-48 or a spherical commercial non-porous silica layer and a γ-Al_2_O_3_ layer).

1. Although a Pd membrane applied to a silicalite-1 layer that was alkali treated by 0.03 M of a NaOH aqueous solution recorded the highest H_2_ permeance at approximately 3 × 10^−6^ mol·m^−2^·s^−1^·Pa^−1^ at 600 °C for all the alkali treated samples, the durability was low.
2. We attempted to decrease the adhesion between the intermediate layers and the SUS by introducing our synthesized mesoporous silica; MCM-48 powders, or commercial spherical non-porous silica particles into the porous SUS supports.
3. The introduction of spherical non-porous silica particles increased the H_2_ permeance of the Pd membrane (membrane thickness: 11 μm) to approximately 10 × 10^−6^ mol·m^−2^·s^−1^·Pa^−1^ at 600 °C, which was higher than the Pd membrane (membrane thickness: 7 μm) following the introduction of MCM-48.
4. Compared with the introduction of MCM-48, the introduction of spherical non-porous silica particles increased the durability of the Pd membrane. These results indicated that commercial spherical non-porous silica particles uniformly occupied the pores of the SUS tubes, which enhanced both the H_2_ permeance and the durability of the Pd membrane.

## Supplementary information

Supplementary information.
