# Supplementary material for: Effects of different silica intermediate layers for hydrogen diffusion enhancement of palladium membranes applied to porous stainless steel support
Source: Sci Rep. 2020 Mar 20;10:5148. doi: 10.1038/s41598-020-62054-3 (PMC7083967; doi:10.1038/s41598-020-62054-3)
Supplement: Supplementary file 1 — Supplementary information. [file 41598_2020_62054_MOESM1_ESM.docx]

Supplementary Information

Effects of different silica intermediate layers for hydrogen diffusion enhancement of palladium membranes applied to porous stainless steel support

Masahiro KATOH*^1^, Tomoe UESHIMA^2^, Masahiro TAKATANI^2^, Hikaru SUGIURA^2^, Kota OMINAMI^2^, Shigeru SUGIYAMA^1^

*^1^Department of Applied Chemistry, Graduate School of Technology, Industrial and Social Sciences, Tokushima University, 2-1 Minamijosanjima-cho, Tokushima-shi, Tokushima 770-8506, Japan*

*^2^Department of Chemical Science and Technology, Tokushima University, 2-1 Minamijosanjima-cho, Tokushima-shi, Tokushima 770-8506, Japan*

**Corresponding author’s e-mail address:* [*katoh@tokushima-u.ac.jp*](mailto:katoh@tokushima-u.ac.jp)


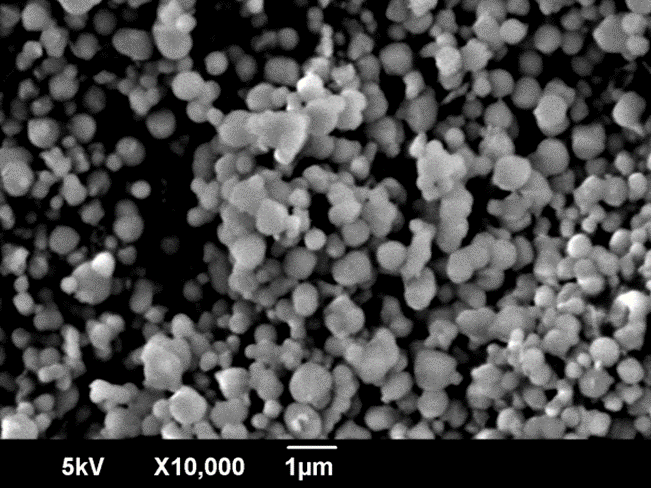


Figure S.1 SEM image of MCM-48 powders.


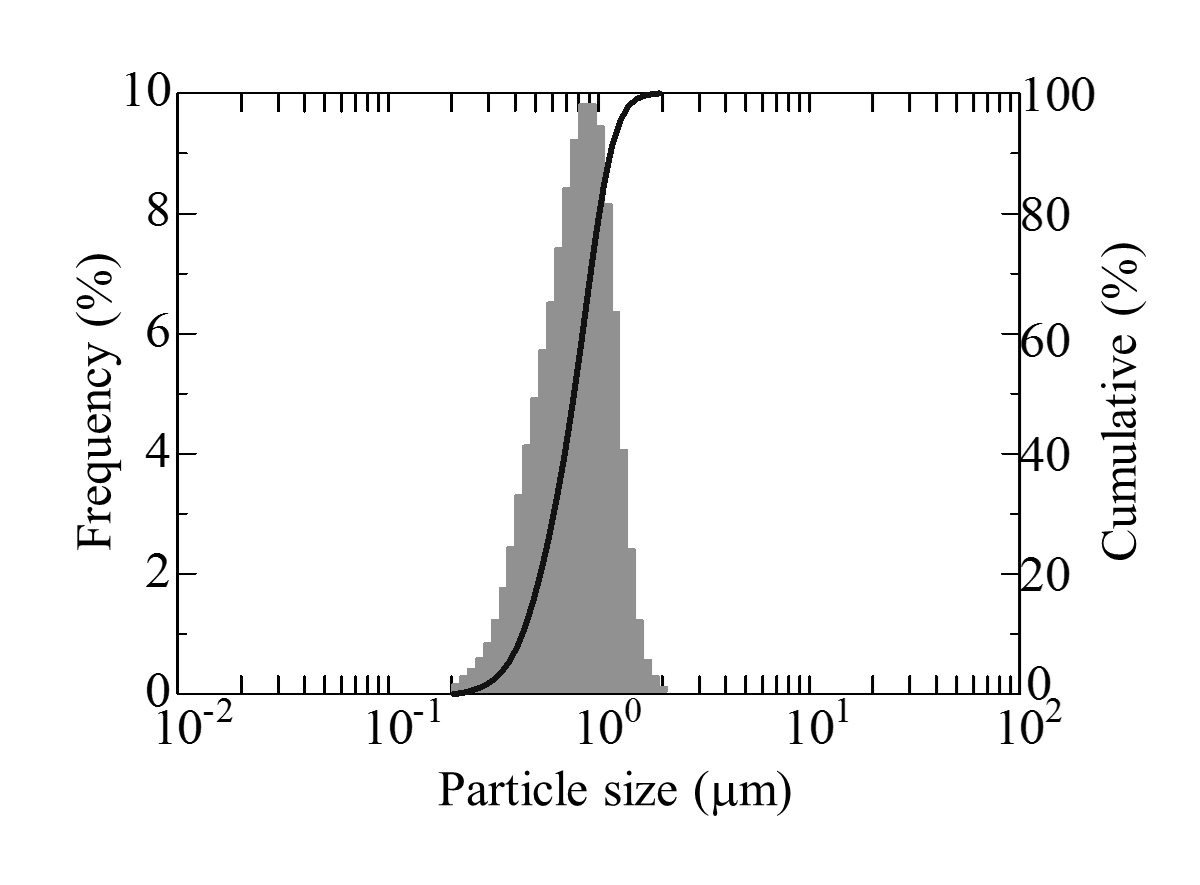


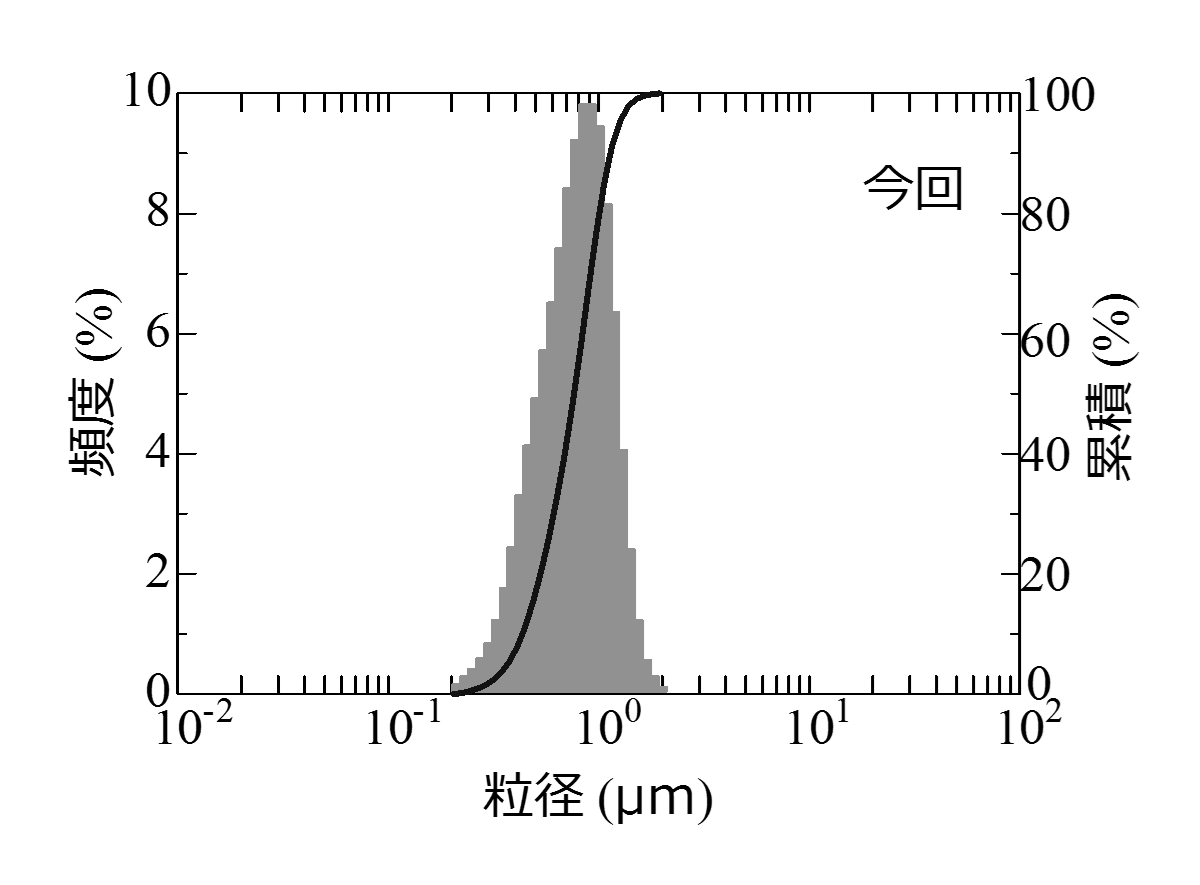


Figure S.2 Particle size distribution of MCM-48 powders


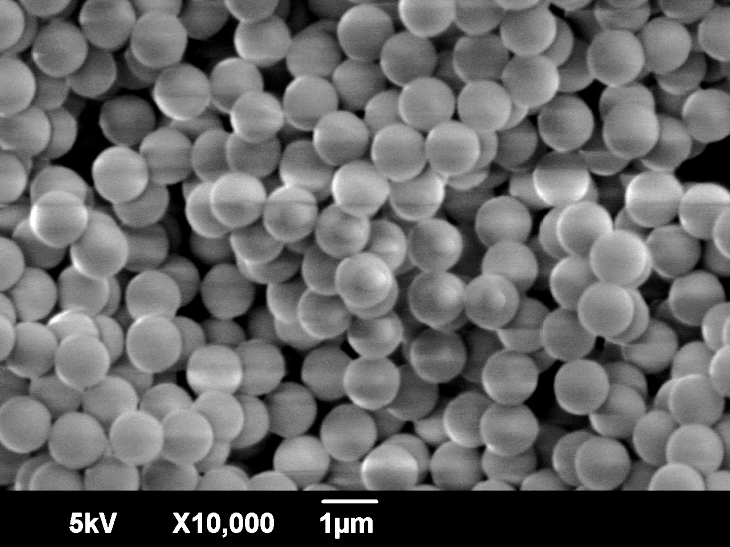


Figure S.3 SEM image of commercial spherical silica particles.


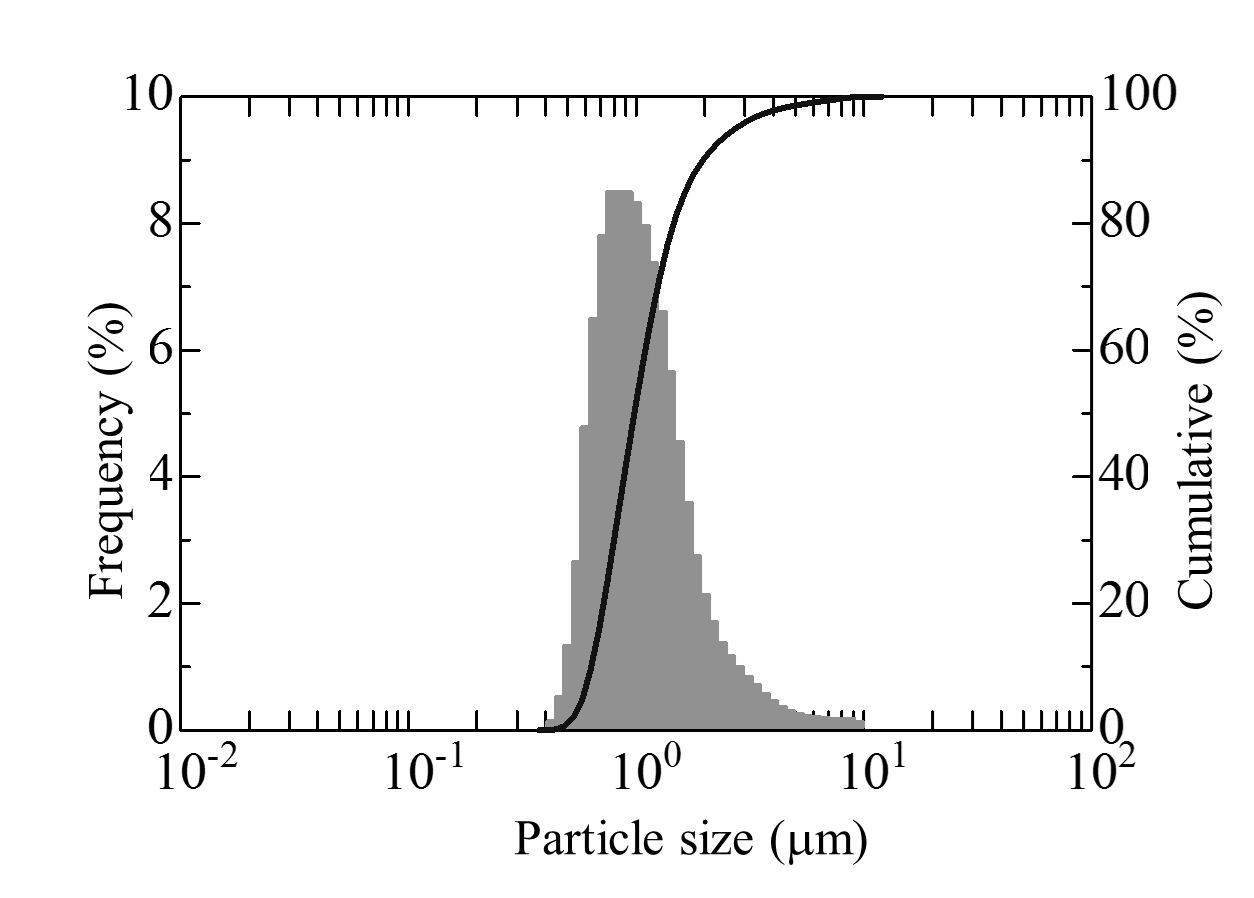


Figure S.4 Particle size distribution of commercial spherical silica particles.
